# AlphaFold2 in biomedical research: facilitating the development of diagnostic strategies for disease

**DOI:** 10.3389/fmolb.2024.1414916

**Published:** 2024-07-30

**Authors:** Hong Zhang, Jiajing Lan, Huijie Wang, Ruijie Lu, Nanqi Zhang, Xiaobai He, Jun Yang, Linjie Chen

**Affiliations:** ^1^ School of Laboratory Medicine, Hangzhou Medical College, Hangzhou, China; ^2^ Key Laboratory of Biomarkers and In Vitro Diagnosis Translation of Zhejiang Province, Hangzhou, China; ^3^ Zhejiang Engineering Research Centre for Key Technology of Diagnostic Testing, Hangzhou, China

**Keywords:** AlphaFold2, deep learning, protein structure prediction, structural biology, disease diagnosis

## Abstract

Proteins, as the primary executors of physiological activity, serve as a key factor in disease diagnosis and treatment. Research into their structures, functions, and interactions is essential to better understand disease mechanisms and potential therapies. DeepMind’s AlphaFold2, a deep-learning protein structure prediction model, has proven to be remarkably accurate, and it is widely employed in various aspects of diagnostic research, such as the study of disease biomarkers, microorganism pathogenicity, antigen-antibody structures, and missense mutations. Thus, AlphaFold2 serves as an exceptional tool to bridge fundamental protein research with breakthroughs in disease diagnosis, developments in diagnostic strategies, and the design of novel therapeutic approaches and enhancements in precision medicine. This review outlines the architecture, highlights, and limitations of AlphaFold2, placing particular emphasis on its applications within diagnostic research grounded in disciplines such as immunology, biochemistry, molecular biology, and microbiology.

## 1 Introduction

**FIGURE 1:**
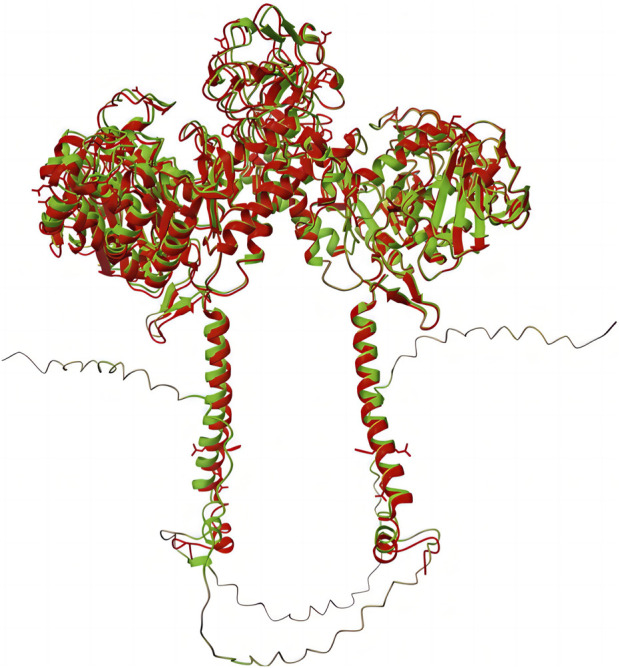
Comparison of X-ray- and AF2-predicted structures of the SLC3A1 homodimer, made using ChimeraX. The X-ray structure is depicted in red, and the AF2-predicted structure is depicted in green. The root mean square deviation between them was 0.333.

AlphaFold2 (AF2), developed by DeepMind, is a modeling method that harnesses the cutting-edge technologies of artificial intelligence and deep learning for predicting protein structures with extremely high prediction accuracy (Figure 1). Rooted in the principle of co-evolution within protein structures, AF2 integrates novel deep learning approaches through the deployment of a suite of trained deep neural network models based on MSA-Transformer, a classical neural network model. These models can generate three-dimensional protein structures with atomic-level precision, informed by both specific amino acid sequence data and information from homologous proteins and multiple sequence alignments (MSAs) (Jumper et al., 2021; Yang et al., 2023). Its outstanding performance at the international CASP14 protein structure prediction competition showcased a significant breakthrough in both speed and accuracy, leading to its decisive triumph (Kryshtafovych et al., 2021). The success of AF2 relies on the accumulation of experimental data on protein structures and the comprehensive research conducted on protein structure prediction. Additionally, the active development community surrounding AF2 ensures a constant influx of fresh talent into the AF2 series, including updates and derivative versions.

**FIGURE 2:**
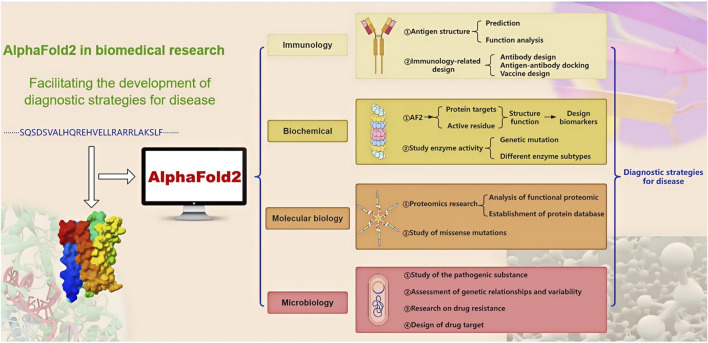
The application of AF2 in the diagnostic strategies for disease. Containing AF2 applications in four areas, the arrows represent progressive relationships.

Proteins play a vital role in physiological processes, and alterations in the structure and function of specific proteins can lead to distinct diseases. Detecting changes in these specific proteins serves as a crucial diagnostic indicator. Proteins are also essential players in the biological functions of pathogenic microorganisms, simultaneously driving disease and influencing treatment strategies. Furthermore, proteins with strong antigenicity not only act as antigens but also serve as potential targets and essential tools in disease diagnosis. Clinical serum antibody detection is one of the many diverse applications of these proteins. The application of specific proteins in disease diagnosis relies on comprehensive research into their unique functions and disease-related changes, involving multiple fields of biology, such as immunology, biochemistry, molecular biology, and microbiology. Since the release of AF2, it has been widely used in various protein research areas. For these studies, numerous excellent reviews have thoroughly explained AF2’s multifaceted functions in biological and medical research, demonstrating its superior performance in predicting protein structures, analyzing mutations, and predicting catalytic and binding sites (Bongirwar and Mokhade, 2022; Paiva et al., 2022; Bertoline et al., 2023). In the meantime, a lot of studies have demonstrated AF2’s robust and exceptional capabilities in investigating disease-related protein structures, functions, interactions, and proteomics. Consequently, research findings utilizing AF2 not only facilitate the development of diagnostic tools and therapeutic drugs, including antibodies and antigens, but also advance our understanding of protein structures, functions, and mutations related to diseases. This helps better understand the impact of specific proteins on the onset and progression of disease, leading to the development of novel disease indicators, targets, detection tools, and treatments (Figure 2). However, there is a lack of comprehensive reviews on AF2’s research in the field of disease diagnosis.

This review aims to comprehensively examine the model architecture, key features, and limitations of AF2. It performs a deep investigation into the extensive applications of AF2 in protein-related research across several disciplines. Finally, this paper briefly touches upon the prospective future development of AF2 and discusses the promotion of basic biological research using AF2 in disease diagnosis.

## 2 AF2

### 2.1 The model structure of AF2

**FIGURE 3:**
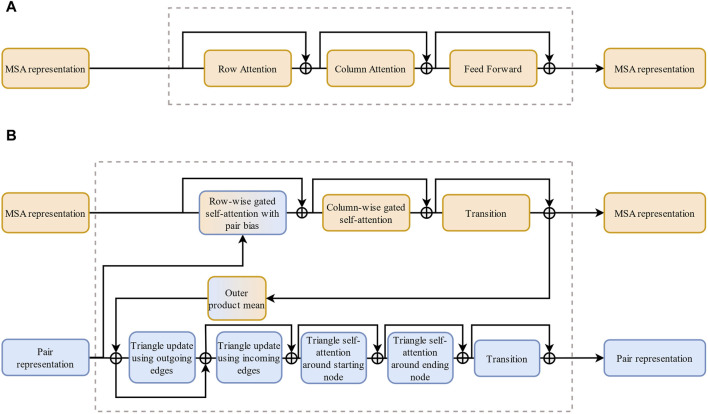
The comparison of MSA-Transformer and Evoformer. **(A)** Architecture of MSA-Transformer. **(B)** Architecture of Evoformer. Compared with **(A)**, a set of modules is added in **(B)** to handle paired information.

AF2 is DeepMind’s foremost protein structure prediction method, distinguished by its utilization of the innovative neural network architecture known as Evoformer. Inspired by the MSA-Transformer (Figure 3) (Vaswani et al., 2017; Rao et al., 2021), Evoformer combines evolutionary mechanisms, physical principles, and geometric constraints inherent in protein structures to yield exceptional protein structure predictions. Evoformer, comprising two sets of MSA-Transformer-based structures, captures information from MSAs and features related to structural constraints between amino acid residues. This dual-focus approach significantly enhances the prediction quality.

At the core of AF2 lies the application of structural information embedded in protein co-evolution (Pazos and Valencia, 2008; Ashenberg and Laub, 2013). MSA is a bioinformatics technique used to align three or more biological sequences, such as proteins, DNA, or RNA. The objective of MSA is to identify regions of similarity that suggest functional, structural, or evolutionary relationships among the sequences. This method arranges the sequences so that homologous residues, which are derived from a common ancestor, are aligned in columns (Prjibelski et al., 2019). By doing so, MSA can uncover crucial information, including conserved sequences and mutation events like point mutations, insertions, and deletions, and can also help infer phylogenetic relationships. MSAs of AF2 sequences are used to extract conservation and covariation information from protein sequences exhibiting co-evolutionary relationships with the target proteins. By integrating this valuable information with structural constraints between amino acid residues, AF2 achieves high-precision and efficient predictions of the target protein’s structure (Yang et al., 2023). Moreover, AF2 incorporates various optimization techniques, such as specific loss functions (Jumper et al., 2021) (e.g., frame point alignment error loss, auxiliary loss and violation loss), a recycling mechanism, self-distillation (Xie et al., 2020), and self-accuracy estimation (Jumper et al., 2021), and other methods to enhance the predictive performance of the model.

**FIGURE 4:**
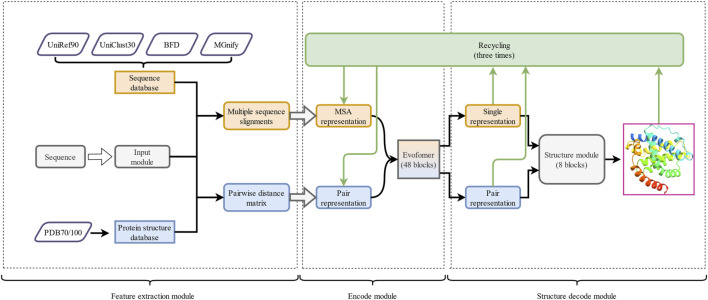
The overall framework of AF2. It is divided into three parts: 1) the feature extraction module, 2) the encode module, and 3) the structure decode module. The arrows represent the execution direction of each output in the runtime architecture. The outputs of the encode module and the structure decode module are continuously optimized through the recycle mechanism.

The comprehensive architecture of AF2 (Tunyasuvunakool et al., 2021; Yang et al., 2023), outlined in Figure 4, comprises three main modules: a feature extraction module, an encoder module, and a structure decoding module. The input module initiates a search for sequences homologous to the template in the sequence database and performs MSA, which reveals similarity and co-evolution information between the protein sequences and is crucial for accurate protein structure predictions. Simultaneously, the input module checks for homologous sequences with known three-dimensional structures and constructs a pairwise distance matrix in the protein structure database to depict the spatial distance between each pair of amino acids. The input module then generates MSA representations and pair representations, which capture co-evolution information and structural constraint features, respectively. The generated MSA pairwise representations are fed into the encode module, which is composed of Evoformer and infers both spatial and evolutionary relationships between proteins using the collected co-evolution information. In the final module, the structure decode module, the output of the encode module is converted into the three-dimensional structure of the target protein. The encoding module and the structure decoding module continuously optimize the predicted structure through the recycling mechanism (Jumper et al., 2021; Yang et al., 2023).

### 2.2 Highlights and limitations of AF2

AF2 utilizes various deep learning training methods combined with efficient search algorithms to collect information from protein sequences and structural data, resulting in more accurate predictions of unknown protein structures.

#### 2.2.1 The neural network architecture adopted by AF2

AF2 uses the Evoformer to learn features of protein sequences and structures from different perspectives. The Evoformer consists of two sets of MSA-Transformer-based modules, which operate on the original MSA and pairwise information and combines a gated mechanism and an attention mechanism to dynamically adjust the network’s output based on the input information (Makkuva et al., 2020). The MSA row-wise gated self-attention mechanism enables the model to capture long-range dependencies in amino acid sequences and protein structures, while the MSA column-wise gated self-attention mechanism allows for element exchange between different species. The Evoformer also learns the geometric constraints inside protein molecules through a triangular self-attention mechanism. The structure decoding module is based on methods similar to MSA-Transformer, encoding residue geometry into a directed reference frame in three-dimensional space (Jumper et al., 2021). AF2 also allows the model to update and optimize its output several times throughout the recycling mechanism to achieve better convergence and stability.

#### 2.2.2 Databases and search algorithms adopted by AF2

AF2 utilizes sequence data from excellent protein sequence databases such as MGnify, Uniclust30, Uniref90, and the Big Fantastic Database, which helps it construct high-quality MSAs (Suzek et al., 2015; Mirdita et al., 2017; Mitchell et al., 2020). The protein structure data are derived from widely recognized databases, including Protein Data Bank (PDB) and PDB70/100 (Steinegger et al., 2019). Such a large amount of amino acid sequence and structure data enables deep learning neural networks to explore various dependencies between protein sequences and structures (Yang et al., 2023), helping to improve the accuracy of AF2 prediction results. AF2 also uses several algorithms, including JackHMMER (Johnson et al., 2010), HHBlits (Remmert et al., 2011), and HHSearch (Steinegger et al., 2019), to significantly improve the search efficiency.

#### 2.2.3 The training methods adopted by AF2

The training set of AlphaFold2 consists of 75% self-distilled data and 25% known structures from the PDB. Self-distillation is a popular method of knowledge distillation that involves the student model learning from the teacher model, thereby enhancing the model’s performance and efficiency. It avoids the complexity and time costs associated with the independent training and optimization of the teacher model in traditional knowledge distillation (Zhang et al., 2019; Xie et al., 2020). During the self-distillation training phase of AF2, the model is initially trained with data from the PDB and then predicts the structures of approximately 350,000 protein sequences in the Uniclust database. These predicted structures are used as data for subsequent training, with the model being retrained on a small subset of random samples in each training cycle. To improve the model’s generalization ability and predictive accuracy, the training data is enhanced through a series of data augmentation processes, including random filtering, MSA preprocessing, and amino acid cropping. Such methods allow the model to make more effective use of limited data and enhance its capability to handle different protein domains and diverse MSA data (Jumper et al., 2021).

#### 2.2.4 The robust AF2 development community

AF2 also boasts a thriving development ecosystem, with DeepMind and researchers in related fields continually updating and expanding on it to meet their investigative needs. For instance, Evans et al. modified AF2 to facilitate predictions of multi-chain complexes, dubbing this enhanced model AlphaFold-Multimer (Evans et al., 2022). Gao et al. built upon AF2 to devise a system, AF2Complex, capable of predicting direct physical interactions between multi-protein assemblies without requiring paired MSA input (Gao et al., 2022). Wayment-Steele et al. employed sequence clustering of protein sequences based on similarity and subsequently applied AF2 to each cluster to predict alternative conformations, a methodology they termed AF-Cluster (Wayment-Steele et al., 2023). Recently, in collaboration with Isomorphic Labs, DeepMind unveiled the latest iteration of AlphaFold, AlphaFold3 (AF3), which, beyond predicting protein-protein interfaces, is capable of forecasting interactions between proteins and nucleic acids and proteins and small molecule ligands, as well as those between antigens and antibodies (Abramson et al., 2024).However, DeepMind is not releasing the AF3 as open source. The multidimensional advancements surrounding AF2 showcase its immense potential across various scientific disciplines.

#### 2.2.5 However, AF2 lacks sufficient predictive ability for the fine structure of proteins

A study by He et al. showed that there are significant differences between the AF2-predicted structures and experimental structures in many aspects, such as the assembly of extracellular and transmembrane domains, the shape of ligand-binding pockets, and the conformation of the transduction binding interface (He et al., 2023).The predicted structure and relative positioning of each domain in AF2 exhibit uncertainty, regardless of the confidence level. This uncertainty can be attributed to several factors (Akdel et al., 2022). One such factor is the presence of indecipherable protein disorder regions in the X-ray data used for AF2 training, which results in the generation of low-confidence, disordered segments in AF2 predictions. Another factor is that some highly confident structural domains are connected by flexible links, leading to errors in the relative positioning of the domains. This uncertainty introduces the possibility of inaccurate results or identifications in structural similarity, structure of pockets, mutational effects, or model construction. These findings underscore the highlight of experimental research in protein structure analysis and emphasize the need for manual inspection and correction of AF2-predicted structures with experimental data. Consequently, the integration of experimental data and artificial intelligence has emerged as a potential solution to addressing these challenges.

During our usage, we observed that AF2 failed to simulate the natural conformation of the receptor-binding domain (RBD) of the SARS-CoV-2 spike protein that “pops out” due to enzymatic cleavage, regardless of whether or not a custom template was provided. We speculate that this limitation may stem from the development of AF2 based on protein structures in aqueous solutions, which are unable to replicate the effects of environmental conditions such as solvent conditions, pH, and ion strength on protein structure (Rey et al., 2023).

### 2.3 Other methods of protein structure prediction

Before the advent of AF2, the first generation of AlphaFold (AF1) had already made significant strides in the field of protein structure prediction by employing deep learning to forecast the distances between protein residues. AF1 constructed a potential of mean force based on these distances, which allowed for the creation of highly accurate protein structures without complex sampling procedures (Senior et al., 2020). Subsequently, AF2 has built upon these achievements by incorporating new neural network architectures and training methodologies. By integrating evolutionary, physical, and geometric insights into protein structures, AF2 has notably increased the precision of predictions, achieving atomic-level accuracy even for proteins without known homologous structures. In addition to AlphaFold, this section will introduce four other protein structure prediction models: Rosetta, RoseTTAFold All-Atom, ESMFold, and RGN2, each with its own distinctive features and strengths (Table 1).

**TABLE 1:** Features, advantages and limitations of 5 different protein structure prediction models.

Method	Feature	Advantages	Limitations
AlphaFold2	A neural network architecture combining attention mechanisms and evolutionary information	1. High accuracy in protein structure prediction 2. Continuous updates and development	1. High computational resources requirements 2. Homologous sequence dependence 3. Lack of fine structure prediction ability
Rosetta	Uses energy functions with fragments, Monte Carlo strategies	High computational efficiency with low search space	1. Limited exploration for intricate topology proteins 2. Low-resolution energy functions
RoseTTAFold All-Atom	Merges sequence-based representations of biopolymers with atomic graph representations of small molecules and covalent modifications	Prediction of proteins, nucleic acids, small molecules, metals, covalent modifications	1. Average accuracy 2. Small training datasets
ESMFold	Utilizes protein language model with training parameters instead of MSA.	1. Faster prediction speed. 2. Efficient exploration of large-scale protein structure space	1. Limited prediction accuracy 2. Less effective with complex structures
RGN2	Uses AminoBERT language model and recurrent geometric network	Prediction of orphan and de novo-designed protein structures	1. Poor prediction with sufficient sequence homologs 2. Hard to predict beta-sheet structures 3. Limited to local dependencies between Cα atoms

#### 2.3.1 Rosetta

Rosetta (Rohl et al., 2004) is a classical *de novo* protein structure prediction method based on fragment assembly, developed by the Baker Lab at the University of Washington, which has had a long-standing impact and wide application in the field of protein structure prediction. The core principle of Rosetta relies on an energy function that utilizes information from fragments of known protein structures, assembling these fragments through Monte Carlo strategies to simulate the natural folding process of proteins, thereby generating conformations close to the native state. This approach ingeniously transforms the continuous conformational space optimization problem into a discrete fragment combination optimization problem, effectively reducing the search space and enhancing computational efficiency.

Nonetheless, Rosetta is accompanied by several drawbacks (Simkovic et al., 2017; Kuenze and Meiler, 2019). 1) When dealing with proteins of high molecular weight or those possessing intricate topologies, the conformational search strategy based on fragment assembly may fall short in thoroughly exploring the complete conformational space. Consequently, this limitation can lead to the omission of the globally optimal solution. 2) Employing low-resolution energy functions, while enhancing computational tractability, inadvertently compromises the precision in depicting detailed interactions.

#### 2.3.2 RoseTTAFold All-Atom

RoseTTAFold All-Atom (RFAA) is a deep learning network that extends the capabilities of conventional protein structure prediction (Krishna et al., 2024; Marchal, 2024). It incorporates the ability to simulate complete biological assemblies, encompassing proteins, nucleic acids, small molecules, metals, and covalent modifications. RFAA merges sequence-based representations of biopolymers with atomic graph representations of small molecules and covalent modifications to predict the three-dimensional structures of these biological assemblies. This enables RFAA to predict the structure of biomolecules more comprehensively, not limited to pure protein systems alone. In terms of protein structure prediction accuracy, RFAA is on par with AF2.

While RFAA has an immediate effect in protein-small molecule binding design and complex biomolecular assembly modeling, its accuracy still needs to be further improved (Krishna et al., 2024). The RFAA’s training set is relatively small, so larger training datasets are needed to improve prediction accuracy for novel protein-small molecule complexes.

#### 2.3.3 ESMFold

ESMFold (Lin et al., 2023; Meng et al., 2023) is a protein structure prediction method built upon pretrained language models capable of directly generating atomic-level three-dimensional spatial structures from a single protein sequence, eliminating the need for multiple sequence alignments or external modeling programs. It employs the extensive pretraining of the ESM-2 protein language model, currently the largest with 15 billion training parameters (Lin et al., 2023), as a replacement for MSA. The predictive performance of ESMFold in terms of structure improved with both the size of the language model and the comprehension of the protein sequence, which exhibited a negative correlation with perplexity. Notably, the prediction speed of ESMFold is one order of magnitude faster than that of MSA-based methods, enabling efficient exploration of large-scale protein structure space.

However, ESMFold is not without its challenges (Lin et al., 2023). 1) The accuracy of ESMFold predictions shows a negative correlation with the perplexity of the sequence, implying difficulty in inferring the structure when the language model struggles to comprehend the sequence. 2) Currently, there is a disparity in the prediction ability of more intricate structures, such as multiple chains or complexes, compared with that of AF2. Further refinement and optimization of ESMFold are required to bridge this gap.

#### 2.3.4 RGN2

RGN2 (Chowdhury et al., 2022) represents an innovative approach to protein structure prediction that utilizes language models and deep learning to directly generate three-dimensional structures from a single protein sequence, eliminating the need for multiple sequence alignments or external modeling programs. The method incorporates AminoBERT, a protein language model, along with a recurrent geometric network to forecast the local geometry of each residue. AminoBERT, employing a Transformer-based architecture, captures latent structural information from unaligned protein sequences. The recurrent geometric network predicts the local geometry of each residue using a rotation matrix, ensuring rotational and translational invariance and avoiding unrealistic torsion angles. Notably, RGN2 excels in predicting the structures of orphan and de novo-designed proteins, which traditionally poses challenges for MSA-based methods.

Nevertheless, RGN2 exhibits certain limitations: 1) When applied to proteins with sufficient sequence homologs to generate multiple sequence alignments (MSAs), RGN2 underperforms compared to AF2 which utilizes MSA for protein structure prediction. 2) Challenges persist for RGN2 in accurately predicting beta-sheet structures from single sequences, particularly for orphan and designed proteins. 3) RGN2 primarily predicts local dependencies between Cα atoms and does not directly consider arbitrary pairwise dependencies across the entire protein structure.

## 3 The application of AF2 in the diagnostic strategies for disease

### 3.1 AF2 in antigen research and design of immunological tool in disease diagnosis

In clinical practice, the immunological assays based on antigen-antibody interactions are an important method for identifying pathogenic agents. Utilizing known antibodies or antigens, we can detect their counterparts in test samples. Concurrently, exploring the structure, functionality, and mutations of pathogenic antigens deepens our comprehension of pathogen traits, supports vaccine creation, and aids in identifying receptors that bind to pathogens. This comprehensive strategy in immunology significantly improves diagnostic accuracy. Presently, AF2 has been effectively employed in a variety of research and design endeavors related to immunology. In this section, we will explore its applications within this field.

#### 3.1.1 Antigen structure prediction and function analysis

AF2 is widely employed in the prediction of antigenic structures in pathogens, analysis of the structural and functional characteristics of antigen proteins, and assessment of the impact of antigenic variations. For example, Hu et al. utilized AF2 to predict the novel fold of the rotavirus glycan-binding domain, which was confirmed through X-ray crystallography (Hu et al., 2022). Veit et al. (2022) used AF2 to predict the structure of the Gp5/M dimer of porcine respiratory and reproductive syndrome virus (PRRSV) and analyzed the heterogeneity of PRRSV Gp5 signal peptide cleavage sites. Both Fang et al. and Yang et al. employed AF2 to predict the structure of the S protein of SARS-CoV-2 and its Omicron variant (Yang et al., 2021; Fang et al., 2023). They investigated the impact of mutations in the S protein on its binding arrangement and affinity to the ACE2 receptor. Yang et al. used AF2 to create a high-precision structural model (pLDDT>70) and compared it with experimental data, considering the root mean square deviation (RMSD) values and amino acid charge properties. The results indicated that the Omicron variant affects the interaction between the RBD region of the S protein and ACE2 without altering the interaction site. Additionally, Fang et al. utilized ColabFold (Mirdita et al., 2022), a protein-protein complex prediction model based on AF2, to analyze the S protein complex with two co-receptors, AXL and LDLRAD3. Based on the predictive complex model, they found that the binding modes of AXL and LDLRAD3 are different: AXL binds to the NTD region of S protein, while LDLRAD3 binds to the RBD region of S protein, and there are competitive binding sites with ACE2. These findings align with their experimental results.

#### 3.1.2 AF2 in immunology-related design

There are numerous applications for immunological study in disease diagnosis and prevention, including pathogen detection, antibody level measurement, immune cell analysis, and vaccine development. These tests require antibodies that can react immunologically with the target antigen, as well as auxiliary anti-antibodies as detection tools. AF2 and its derivative models possess the capability to predict protein structures and protein-protein docking. Numerous experiments have demonstrated that AF2 can accurately predict vaccine and antibody structures, as well as optimize antibody-antigen complexes. This highlights its potential in designing tools for immunological detection.

##### 3.1.2.1 Antibody design

Antibodies serve as critical tools for immunological detection. Their ability to bind to antigens largely relies on the topological complementarity between the variable domain of antibodies and the spatial structure of antigen epitopes (Graham et al., 2019). Therefore, accurate identification of the antibody structure and a precise understanding of the antibody-antigen (Ab-Ag) interface (i.e., the antibody epitope) are essential for antibody design (Sela-Culang et al., 2013; Guest et al., 2021; Hummer et al., 2022). Due to the superior performance of AF2 in predicting protein structures, it has been used in several studies to predict antibody structures and epitopes.

There are two major obstacles in predicting antibody structures: 1) determining the relative orientation of the heavy chain (Vh) and light chain (Vl) domains and 2) predicting the complementary determining regions (CDRs), especially highly variable and conformationally diverse CDR-H3 loop structures (Jaszczyszyn et al., 2023). Polonsky et al. achieved highly accurate predictions of 50% of the positions within the Fab region of 222 antibodies using AF2, with an average TM-score of 0.83 for individual Vh and Vl (Polonsky et al., 2023). This not only implies identical folding but also signifies very close proximity between the predicted and native structures. Ruffolo et al. tested the performance of AF2 and AlphaFold-Multimer in antibody structure prediction (Evans et al., 2022; Ruffolo et al., 2023) and found that AlphaFold-Multimer can accurately predict the backbone structure of antibodies, the relative orientation of Vh and Vl, and the CDR loop structure. For the relative orientation of Vh and Vl, they calculated the orientation coordinate distance (OCD) (Marze et al., 2016) of the predicted models to determine the accuracy of the relative orientation between Vh and Vl in the predicted models. The results indicate that the Fv (variable region of antibody) structure predicted by AlphaFold-Multimer has an OCD of 4.18, which is within one standard deviation of the native structure. Moreover, AlphaFold-Multimer demonstrated sub-angstrom accuracy in predicting the CDR1 and CDR2 loop structures, and for CDR3, it exhibited greater prediction accuracy and novel predicted structures compared to many other models, demonstrating superior performance in predicting antibodies such as the PDB identifier 7N3G. AF2 performs best in predicting the CDR structures of nanobodies, as it considers various structural arrangements during the training process, giving it an advantage in predicting the secondary structures of nanobodies. Although both AlphaFold-Multimer and AF2 can predict the structure of antibody CDRs, their ability to predict CDR-H3 loop structures is still insufficient. Continuous updates to AF2 may improve this issue in the future.

The advancement of deep learning methods has allowed researchers to work toward enhancing the accuracy of antibody epitope prediction through the integration of models that combine sequences and structures and incorporate both local and global features (Zeng et al., 2023). Researchers have leveraged AF2’s remarkable monomer protein structure prediction capabilities to forecast antibody epitopes, utilizing the predicted antibody structure model as input data for the prediction system (Desta et al., 2023a; Desta et al., 2023b). Desta et al. devised a method for antibody epitope prediction known as PIPER-Map (Desta et al., 2023b). This approach utilizes AF2 to anticipate antibody structures and employs the docking program PIPER, which is based on fast Fourier Transform (FFT), to perform docking between the antibody models and antigens. The docking results are subsequently ranked for analysis. Studies have shown that this method predicts antibody epitope structures with excellent accuracy, with the AF2 predictions comparable to those based on existing antibody crystal structures. In addition, Desta et al. reviewed the advanced antibody epitope localization software ServerClusPro AbEMap Web Server and investigated the effectiveness of predicting antibody epitopes using the AF2 prediction model as input (Desta et al., 2023a). The results indicated that the antibody epitope predictions generated by AF2 were similar to those generated based on established antibody structure templates, with improved predictive power for partial antibody epitopes such as PDB ID 2W9D compared to X-ray structures. Notably, the performance of AF2 for antibody epitope prediction using existing antibody templates was inferior to that achieved without utilizing antibody templates for prediction.

##### 3.1.2.2 Optimization of antigen-antibody docking models

Antigen-antibody binding serves as the foundation for immunoassays and holds significant value in medical and immunological research. However, the current challenge lies in achieving effective antigen‒antibody docking, and a universal solution to this problem remains elusive (Hogues et al., 2018). Despite these obstacles, AF2 can make robust predictions of protein-protein binding, and it has been successfully used to predict structural aspects of antigen-antibody docking and assess the outcomes of the predictions.

In a study by Yin et al., the ability of AF2 to predict antigen-antibody docking was scrutinized using over 400 nonredundant antigen-antibody complexes (Yin and Pierce, 2024). Their findings indicated that the their than-latest version of AlphaFold, v.2.3, has a higher prediction success rate compared to the previous version, v.2.2. Additionally, the updated AlphaFold demonstrated increased efficacy in predicting nano antigen-antibody docking, underscoring the potential of AF2 in identifying antigen-antibody docking structures. This research emphasized that the accuracy of AF2 can be improved by optimizing the framework or model, enhancing sequence information within the MSA, and establishing a positive correlation between subunit prediction accuracy and the success rate of antigen-antibody interaction predictions. Consequently, the modification of AF2’s architecture, particularly the structural module, holds promise for augmenting prediction accuracy by integrating contemporary factors (Abanades et al., 2023; Ruffolo et al., 2023) that enhance antibody prediction precision, potentially refining AF2’s overall predictive capabilities.

Gaudreault et al. (2023) used AF2 to augment the predictive accuracy of antigen-antibody docking structures, refining the expected docking models and improving early success rates. They employed standardized pLDDT and pTMscore (Z_pTMscore_ and Z_pLDDT_) to compute a composite score, the AF2Composite score, which measures the confidence levels associated with these docking models (Eq. 1). The experimental results demonstrate the practicality, simplicity, and efficacy of this scoring method, which is free from the constraints of a specific physical methodology and remains uninfluenced by any subjective biases introduced during training or calibration. Notably, the correlation between the score and the experimentally observed docking structure strengthened with increasing quality of the predicted docking models. For instance, when *R* ^2^ < 0.4 (indicating poor mutual correlation between pLDDT and pTMscore), the correlation is significant only for models of acceptable quality. For models exhibiting superior prediction quality, the score proves instrumental in elevating the ranking of true positives within the predictive structure, thereby enhancing the discriminatory ability of these prediction models in the negative/positive classification of antibody-antigen docking.

$${AF2}_{Composite}={Ζ}_{pLDDT}+{Ζ}_{pTMscore}$$ (1)

#### 3.1.3 Vaccine design

The vaccine development for respiratory syncytial virus (RSV) has demonstrated the importance of structure-based vaccine design (Graham et al., 2019). Using AF2 to predict protein structures could aid in structure-based design, potentially overcoming difficulties faced in previous vaccine development work.

Currently, various antibodies targeting the hemagglutinin (HA) stem region have been identified as neutralizing antibodies against influenza B virus (IBV). Therefore, vaccines designed based on HA can broadly prevent IBV infection. Zheng et al. used AF2 to design a hemagglutinin stem cell vaccine specific to IBV, named “B60-Stem-8071” (Zeng et al., 2022). They used AF2 to predict the vaccine’s structure and screened for vaccine sequences that could correctly fold and maintain the natural conformation of the HA stem region in prokaryotic systems. Additionally, to enhance the stability of the HA stem region structure and improve the immune response against HA vaccine *in vivo*, they rationalized and engineered the epitope linker of the neutralizing antibody CR8071 using AF2, connecting the optimized structure to the vaccine, allowing it to target the CR8071 epitope.

### 3.2 AF2 in biochemical studies

#### 3.2.1 Development of auxiliary protein targets and biomarkers

Proteins that perform crucial functions in vital life processes, such as enzymes, receptors, and ion channels, serve as significant targets for biochemical detection and drug therapy. While protein-protein interaction has been identified as a new path to discover protein targets (Liu et al., 2024), the structure and function of novel proteins are often difficult to determine. Studying protein targets with AF2 can not only predict the interaction between proteins to find protein targets but can also improve the understanding of protein structure and function, accelerate drug design, and contribute to advances in biology and medicine. Gόmez-Marίn et al. used AF2 to predict the structure and interaction domain of high mobility group 20A (HMG20A) and PHD Finger Protein 14 (PHF14) and found that they form a stable nuclear complex through coiled-coil domain interactions, identifying them as potential protein targets (Gómez-Marín et al., 2022). It can affect important biological processes, such as epithelial–mesenchymal transition and the TGF and Hippo signaling pathways.

Transmembrane proteins are recognized as significant targets in drug design. Hegedűs et al. reported that AF2 can accurately predict the structure of transmembrane proteins, highlighting the usefulness of AF2 in transmembrane protein studies (Hegedűs et al., 2022). This study provides valuable information for research into the ability of transmembrane proteins to correct structural errors, discover new conformational states, and simulate kinetic processes. Loring et al. used AF2 to predict the structures of different subtypes of resistance to inhibitors of cholinesterase 3 (RIC-3) (Loring, 2022). Based on these predicted structures, they analyzed how RIC-3 interacts with the alpha7 nicotinic receptor (α7 nAChR) subunits and promotes the folding and assembly of the α7 nAChR into the final conformation and subsequently proposed two possible models for the interaction between RIC-3 and α7 nAChR.

The function of these critical proteins often relies on their essential active residues. When the structures of these residues change, it can lead to alterations in protein function and concentration, which frequently preludes the onset of disease. Several studies have utilized AF2 to gain insight into protein function, uncover protein interactions, and identify crucial protein active sites, contributing to the advancement of disease diagnosis. Freeman et al. (2023) used AF2 to construct a structural model of the nuclease Ankyrin Repeat and LEM Domain Containing 1 (ANKLE1) and analyze its key active residues. The results indicated that the mutation of each of these residues impaired enzyme activity. ATG8/LC3 is the key protein involved in the autophagic process, and the ATG8-interacting motif/LC3-interacting region (AIM/LIR) facilitates the binding of ATG8 to autophagy cargo receptors and adaptors (Fracchiolla et al., 2017). Ibrahim et al. used AlphaFold-Multimer to analyze the spatial structure of the ATG8/LC3 protein family and accurately predicted the pockets formed by both typical and atypical AIM/LIR within the family (Ibrahim et al., 2023). The functions and effects of these pockets in the autophagy pathway were further analyzed in this way. They also utilized three pathogen virulence factors to demonstrate that AlphaFold-Multimer could effectively identify motifs from a variety of AIMs that bind ATG8.

Proteins can serve as molecular biomarkers and are frequently utilized for early disease screening, diagnosis, prognosis assessment, individualized treatment plan formulation, and prediction of adverse drug reactions (Aronson and Ferner, 2017). The development and screening of characteristic molecular biomarkers are crucial for determining the specificity and accuracy of molecular disease diagnosis (Molinski et al., 2020). Proteins with specific modifications during disease development, along with their crucial active residues, can serve as biomarkers of disease. Consequently, AF2’s ability to investigate protein targets and their associated residues could significantly contribute to biomarker development. Zhuo et al. used next-generation sequencing (NGS) to determine the amino acid sequences of the immunoglobulin and T-cell receptor V-(D)-J region in bone marrow samples of 47 children with precursor B-cell acute lymphoblastic leukemia (pre-B-ALL), and they used AF2 to predict the protein structure based on the results (Zhuo et al., 2023). They extracted the immunoglobulin heavy chain gene (IGH) CDR3 consensus sequence with rod-shaped α-helix structure similarity from the predicted protein structure as an IGH rod-shaped tracker. They further validated the predictive value of the IGH rod tracker using published IGH data from an additional 203 children with pre-B-ALL. They found that the prognosis for children who tested positive for NGS-IGH was poorer than that of those who tested negative, and they also found that the protein structure encoded by the IGH CDR3 was consistent across all NGS-IGH (+) samples. These findings suggested that the sequence could serve as a marker for monitoring minimal residual disease in children during treatment.

#### 3.2.2 Characterization of effect of mutation on enzyme activity and the difference of enzyme activity among different subtypes

Enzyme activity and enzyme metabolites are two crucial markers in biochemical detection. Alterations in either can signify changes in associated physiological indicators and the onset of related diseases. AF2 has been widely used to study the effects of structural differences and variations in enzyme activity and enzyme metabolites, providing a basis for biochemical detection and mechanism interpretation of enzymes involved in vital activities. Aminolevulinic acid synthase (ALAS), a key regulator of catalytic heme synthesis during the initial steps of key enzymes (Taylor and Brown, 2022; Freeman et al., 2023), can carry a mutation in the extended C-terminus of the erythroid isoform (ALAS2) that impacts its ability to efficiently catalyze heme synthesis, resulting in increased risk of X-linked protoporphyria. Hunter et al. used AF2 to study the structural differences among various ALAS variants, as well as the mechanism by which the C-terminal extension of ALAS controls the rate of porphyrin synthesis (Hunter and Ferreira, 2022). They predicted the structure of six mammalian ALAS subtypes and compared the predicted structure of ALAS1 with that of ALAS2. They found that the CXXC motif and the heme regulatory motifs (HRM) 4 and 5, which extend the C-terminus of ALAS, regulate ALAS activity. Their analysis of the ALAS1 structure revealed that the CXXC motif forms disulfide bonds in its oxidized state, causing HRM4 and HRM5 to fold and thereby preventing their inhibitory effect. The CXXC motif is reduced to expose HRM4 and HRM5, inhibiting excessive heme synthesis. Furthermore, the different positions of HRM4 and HRM5 in ALAS2 compared to those in ALAS1 prevent the closure of HRM4 and HRM5 at the extended C-terminus, resulting in the inability of the cellular redox state to regulate excessive hemoglobin concentrations.

Wiedemann-Steiner syndrome (WDSTS) is a neurodevelopmental disorder caused by *de novo* mutation of lysine methyltransferase 2A (KMT2A, a multidomain histone methyltransferase) (Jones et al., 2012). Reynisdottir et al. (2022) reported that the onset of WDSTS was closely related to the loss of the ability to recognize and bind unmethylated CpG in the CXXC domain of KMT2A due to variation. They used AF2 to predict the structure of various variations in the CXXC domains and established a high-precision classification scheme for the effects of these variations. All possible missense variations in the CXXC domain were predicted, and the variants were classified into three types based on the predicted results: no effect, damage to DNA binding, or non-folding of the domain. This allowed for the accurate determination of potential pathogenicity and effects on function that the missense variations in the CXXC domain have, thereby providing a reference resource for disease diagnosis.

### 3.3 AF2 in molecular biology studies

#### 3.3.1 Proteomic research

Proteomic research involves the qualitative and quantitative study of proteins with the aim of understanding the mechanisms by which they carry out their physiological activities and exploring disease process and pathogenicity to guide diagnosis and novel drug development (Hanash, 2003; Aslam et al., 2017). Technological advancements have allowed proteomics to play a pivotal role in disease diagnosis. By comparing protein expression and functional changes between control and case groups, researchers can study specific protein characteristics associated with disease. This aids in early disease diagnosis and prognostic monitoring while also allowing for the analysis of individual protein variations to inform personalized diagnosis and medical treatment. However, due to the dynamic range and large scale of the proteome, traditional mass spectrometry methods still face challenges in terms of data acquisition and verification. With its strong data processing and mining capabilities (Zhang et al., 2014), AF2 is able to predict the three-dimensional structure of single-chain proteins as well as of protein complexes, making it particularly useful for proteomic studies.

##### 3.3.1.1 The function of AF2 as a proteomic tool

Functional proteomics is the study of protein-to-protein or protein-to-nucleic acid interactions in a specific time and space, focusing on a functional subgroup of proteins within a cell. AF2 has been widely used in functional proteomics research due to its excellent predictive speed and accuracy, enabling large-scale research and cluster analysis of protein functions (Huang et al., 2023). By searching for proteins containing the Z-DNA/Z-RNA binding protein (Zα) domain in the AF2 predictive structure database, Bartas et al. identified 185 proteins that may bind to Z-DNA/Z-RNA and play an important role in a variety of cellular processes (Bartas et al., 2022). Huang et al. (Huang et al., 2023) selected 15 genes with a length greater than 100 bp from the deaminase family, predicted their structures, and compared them with those in the AF2 database. Based on the comparison results, a similarity matrix was generated, and a structure tree was constructed to perform a cluster analysis on the deaminase family to elucidate the structural and functional differences among different deaminases within the family. Al-Masri et al. (2023) analyzed known protein kinase structures in the AlphaFold protein structure database to predict the specific structures of several protein kinases, subsequently using Smina to perform molecular docking on protein kinase crystals matching the protein kinase structure to evaluate the effectiveness of AF2 in virtual filtering. The results show that AF2 can effectively simulate kinase active sites that are highly characteristic of conformational states, providing a foundation for the study of protein kinase pathogenicity and the development of new drugs based on kinase active sites.

##### 3.3.1.2 Establishment of protein database

AF2 provides a high-quality and efficient method for generating and analyzing large-scale protein structure databases, which is crucial in proteomic research (Domon and Aebersold, 2006; Fremdling et al., 2022). The construction of a protein information database is an essential step that significantly increases the speed of protein identification and the development of mass spectrometers. AF2 can be used to construct large-scale protein structure databases, providing rich and reliable protein structure resources for proteomic research and facilitating the establishment of relevant datasets for mass spectrometers. Varadi et al. created a comprehensive, open access database of high-accuracy protein structure predictions (Varadi et al., 2022). AlphaFold database contains a considerable number of high-accuracy protein structure prediction models, offering valuable resources for biological research. Hekkelman et al. used small molecules and ions in experimentally determined protein structures to “transplant” the protein model in the AlphaFold protein structure database, thereby establishing the AlphaFill database (Hekkelman et al., 2023). The database contains 12,029,789 “transplant” results of 995,411 AF2 models, providing relevant validation indicators and visual interfaces, enriching model information in the AlphaFold database, and offering researchers clues to new protein function hypotheses. Consequently, AF2 can deliver high-quality and efficient generation and analysis methods for the construction of large-scale protein structure databases, providing more possibilities for proteomic research and mass spectrometer development.

#### 3.3.2 AF2 in the study of missense mutation on protein structure and function

Missense mutations can serve as biomarkers in clinical molecular biology tests. These mutations may alter the amino acid sequence and structure of proteins, thereby affecting their function and pathogenicity. Many studies have utilized AF2 to predict and compare the structures of normal and mutated proteins, thereby revealing the mechanisms and effects of missense mutations.

Wang et al. (2023) reported a novel mutation in the lysosomal membrane structural protein (LAMP2) gene and used AF2 to predict the three-dimensional structures of wild-type and mutant LAMP2. They found that the mutant LAMP2 is composed of only six amino acids and that it is unable to form functional peptides or proteins, confirming that LAMP2 deficiency is caused by this mutation. The LMNA gene encodes the lamin A/C protein, which is involved in the construction of nuclear membranes, and mutation of LMNA results in a series of lamin diseases. Chang et al. (2023) used AF2 to predict the spatial structure of the lamin A/C mutant protein and found an interruption in the alpha-helix region. They used this protein structure to visualize the impact of the mutation on protein morphology and interaction compared to the wild-type protein. Finally, they used AF2’s predictions to elucidate the mutation’s pathogenicity at the protein level, revealing the function of different protein domains and potential therapeutic targets.

Despite great progress in AF2’s ability to predict the structure of mutant proteins, some researchers have pointed out the limitations of AF2 in predicting the impact of missense mutations on protein stability. Buel et al. emphasized these limitations by comparing AF2-predicted models of wild-type and mutant structures of three protein domains to the experimentally determined structures of the wild-type proteins (Buel and Walters, 2022). This comparison revealed that the predicted models did not accurately reflect the structural changes and functional losses induced by the mutations. To address this issue, researchers have developed AF2 prediction models to deduce the structure and stability of proteins after mutation. For example, Iqbal et al. developed a predictive model, protein stability (PROST), that can estimate the changes in protein stability caused by single-point missense mutations (Iqbal et al., 2022). In two blind test datasets, PROST outperformed the other models in terms of predictive performance, achieving the highest Pearson’s correlation coefficient and the lowest root mean squared error. This indicates that PROST has good accuracy and can serve as an important tool in the prediction of the three-dimensional structure of mutant proteins. Cheng et al. developed a model based on AF2, called AlphaMissense (Cheng et al., 2023), that was fine-tuned based on AlphaFold 2.3.0 using human and primate variant frequency data as weak labels and avoiding circularity arising from the use of manual annotations. AlphaMissense can simulate all possible single amino acid mutations and can distinguish 89% of missense variants as likely pathogenic or likely benign.

AF2 not only predicts the structural changes in proteins resulting from missense mutations but also analyzes the impact of these changes on protein function. It generates various models to predict the stability changes caused by missense mutations and the likelihood of pathogenicity. AF2 therefore plays a crucial role in missense mutation research—it can not only explain the pathogenic mechanisms of missense variants but can also identify missense mutations with potential clinical significance, providing biomarkers for disease diagnosis.

### 3.4 AF2 in pathogenic microbiology research

Pathogenic microorganisms play a crucial role in laboratory disease diagnosis. Factors such as biological characteristics, drug resistance, and variant typing all affect the pathogenicity of microorganisms, the symptoms of disease, and the effect of drug treatment. For example, the major resistance mechanism in MRSA is via the acquisition of the gene mecA, which encodes the protein PBP2a. MecA, however, has a significantly low affinity for β-lactam, which makes all currently available β-lactam drugs largely ineffective for the treatment of MRSA (Peacock and Paterson, 2015). The key proteins involved in the pathogenic process of microorganisms are also important targets for drug development and screening.

Traditional laboratory diagnostic methods for pathogenic microorganisms (Rajapaksha et al., 2019) include culture and isolation, biochemical and serological detection, and immunological and nucleic acid assays. However, these methods have significant limitations, such as extended diagnostic time, low detection rate, inability to fully interact with *in vivo* infections, and inability to culture certain microorganisms. The advancement of cutting-edge biological theories and technologies, such as mass spectrometry (Schubert and Kostrzewa, 2017) and molecular diagnostics (Lai and Stayton, 2015; Visconti et al., 2017; Yasemin et al., 2019), coupled with the progress of artificial intelligence (Jumper et al., 2021; Tunyasuvunakool et al., 2021), makes it possible to examine clinical pathogenic microorganisms based on studies of the structure, function, and distribution of microbial proteins. To date, many studies have used AF2 to determine the pathogenicity, microbial resistance, and potential drug targets of microorganisms.

#### 3.4.1 Study of pathogenic substances

Considering its direct impact on clinical manifestations and disease progression, studying the pathogenicity of microorganisms is key to revealing the core pathogenic mechanisms and promoting the identification and targeted treatment of pathogens. Use of AF2 in the in-depth analysis of the structural and functional properties of these key proteins that considers the composition of a variety of pathogenic proteins and biomolecules is driving the rapid development of the detection and treatment of pathogenic microorganisms. With the assistance of AlphaFold-Multimer, Le et al., 2023 predicted the structural model of the outer membrane lipoprotein Tle3, its cognate immune protein Tli3, and their immune complexes of adhesively invasive *Escherichia coli* (AIEC) and optimized the model through molecular replacement. They found that a β-lamellia stacking region in the C-terminal extension domain of Tli3 intercalates into the active cleave of Tle3, suggesting that Tli3 physically blocks Tle3 from contacting its substrate and thereby inhibits its phospholipase A1 activity. They used similar methods to predict the mode of interaction between Tle3 and VgrG, a protein constituting the spinous process of the type VI secretion system, and found a potentially specific interaction between the N-terminal loop of Tle3 and the C-terminal transthyretin-containing domain of VgrG. This provided vital structural and biochemical information for understanding the function and mechanism of type VI secretion system effectors and immune proteins in AIEC, which is of great significance for revealing the pathogenesis of AIEC and identifying new therapeutic targets. These findings will aid in the development of new anti-AIEC drugs or diagnostic reagents, thereby enhancing the efficiency and accuracy of clinical microbiology.

#### 3.4.2 Assessment of genetic relationships and variability

AF2 has been used to analyze the differences in the protein structures of various strains or phages and evaluate their genetic relationships and variability. This has proven beneficial for the classification and identification of different species of microorganisms, providing a reference for epidemiological surveillance and control. Goulet et al. employed AF2 to predict three-dimensional models of the components of the adhesion apparatus of two bacteriophage types, OE33PA (Jaomanjaka et al., 2018) and Vinitor162 (Philippe et al., 2020), that infect *Oenococcus oeni* (Goulet and Cambillau, 2021). Based on the known architecture of the phage adhesion apparatus, a topological model was reconstructed. OE33PA possesses an evolved distal tail protein (Dit) (Veesler et al., 2010) and an exotic receptor-binding protein (RBP), composed of two domains similar to the RBPs of different phages, and forms a chimeric structure. By contrast, Vinitor162 has a long tail-associated lysozyme protein (Tal) that is rich in carbohydrate-binding modules (CBMs). This finding suggests distinct infection mechanisms between OE33PA and Vinitor162: OE33PA employs a dual binding strategy involving its Dit-CBM and RBP head domain to engage receptors on the host cell wall for entry, whereas Vinitor162 utilizes a multipoint attachment mode through its Tal-CBM and RBD to infect host cells by interacting with receptors on the host cell wall. Monzon et al. used AF2 to predict structures lacking known adhesion domains in more than 6,500 credible fibrillar adnexins and identified 24 potential novel families of adhesion protein domains, 15 of which showed structural similarity to known adhesion domains. This contributes to the discovery of novel bacterial interaction mechanisms (Monzon and Bateman, 2022).

#### 3.4.3 Research on drug resistance

AF2 can be used to predict mutation-induced changes in the protein structure of microorganisms as well as unreported protein structures, thereby assisting in the analysis of microbial resistance mechanisms. Multidrug-resistant *Acinetobacter baumannii* (*A. baumannii*) is one of the leading pathogenic causes of severe nosocomial infections. *A. baumannii* CipA has been identified as a plasminogen-binding and complement-inhibitory protein that plays a significant role in its immune evasion process. The use of AF2 in the structural prediction of CipA aptly explained the results obtained from several CipA variants (Ries et al., 2022). According to the structural prediction of AF2, replacing the glutamic acid (E) at position 360 with a proline (P) will induce a significant structural change in the C-terminal region of the DUF4377 domain, and the hydrogen bond pairing of the adjacent β-fold is completely lost. This change greatly inhibits the ability of CipA to interact with complement factor I, which will provide potential targets for new therapeutic interventions.

Some researchers have also successfully predicted unresolved structures using AF2. Willems et al. used the AF2 algorithm to solve domain structures that were not resolved in the previously reported *Plasmodium falciparum* Chloroquine Resistance Transporter (PfCRT) protein 7G8 isoform cryo-EM structure (Willems et al., 2023). When cryo-EM was used to analyze the 7G8 isoform of PfCRT, many of the N- and C-termini, as well as the cytosolically disposed “loop 2” connecting TM helices 2 and 3, were not resolved, presumably due to masking by bound F’ (ab) (a type of incomplete F (ab) fragment) used in solving the cryo-EM structure and/or the intrinsic flexibility of these regions. They then performed energy minimization through Monte Carlo molecular dynamics simulations, revealing additional structures for the previously unresolved N- and C-termini. These results are crucial for understanding the structure and function of PfCRT, the mechanism of chloroquine resistance, and the development of novel second-tier drug therapies active against chloroquine-resistant malaria. The above examples further highlight the significant application and value of AF2 in biomedical research.

#### 3.4.4 Design of drug targets

In recent years, the emergence of drug-resistant strains has gradually diminished the therapeutic effect of antibiotics on pathogenic microbial infections (Davies and Davies, 2010). To address these new challenges in anti-infection treatment and drug screening, some studies have employed AF2 to study the structure and function of proteins related to pathogenic microorganisms. This has advanced research on potential drug targets, the development of antibacterial drugs, and the screening of drugs and antimicrobial peptides. Madi-Moussa et al. used AF2 to predict the structure of Lacticaseicin 30, a rare gram-positive bacteriocin that inhibits gram-negative bacteria (Madi-Moussa et al., 2022). They found that it primarily consists in the five helical segments and contains regions and amino acids involved in anti-gram-negative activity. By studying the antimicrobial activity of a series of shortened variants or those containing point mutations in the five helical segments, they mapped these regions and the amino acids involved in inhibition. These experiments showed that at least two helical segments of the N-terminal region are required for Lacticaseicin 30 inhibition of gram-negative bacteria, which will aid in the design of additional Lacticaseicin 30 variants as potential drugs treatments of gram-negative bacterial infection. Alotaibi et al. screened a series of drug target proteins against *Vibrio* by gene alignment and used AF2 to predict the three-dimensional structure of 2,3-bisphosphoglycerate-independent phosphoglycerate mutase, a drug target protein (Alotaibi et al., 2023). Furthermore, some effective inhibitors were identified through virtual screening (Panwar et al., 2024) and molecular docking studies (Pinzi and Rastelli, 2019), and their binding stability with target proteins was verified using molecular dynamic simulations (Koirala et al., 2024).

## 4 Conclusion and future perspectives

Proteins play a crucial role in disease diagnosis. They serve as diagnostic indicators and detection tools, contributing to accurate diagnosis, disease prevention, and personalized medicine. AF2, a deep learning-based protein structure prediction model, achieves remarkable accuracy and rapid protein structure predictions through its unique principles and architecture. As such, it has applications in diverse areas of protein research (Yang et al., 2023).

AF2 significantly contributes to disease diagnosis by predicting antibody structures for immunological tests and vaccines, verifying antigen-antibody affinity, and aiding in diagnostic tool design. It’s used to predict structures of disease-related proteins, enhancing our understanding of their structural, functional, and activity changes. These insights form the basis for improving diagnosis, prevention, and treatment. AF2 also analyzes key enzyme variations during disease progression, establishing diagnostic criteria. It supports proteomic data analysis, database creation, and research. Additionally, AF2 assesses missense variation impacts, aiding in biomarker design. It studies pathogenic substances’ functions, drug resistance, and classification by microorganisms, aiding in accurate infection diagnosis and drug target development.

AF2 is highly adaptable and presents with unlimited potential for extensive application in several biological fields. Various prediction models based on AF2 with expanded functions have emerged, examples of which include AlphaFold-Multimer (Evans et al., 2022; Yin et al., 2022; Ibrahim et al., 2023), AF2Complex (Gao et al., 2022), ColabFold (Mirdita et al., 2022), and AlphaMissense (Cheng et al., 2023). Future versions of AlphaFold may prioritize the optimization and refinement of its architecture to enhance its predictive ability and broaden its functionality (Abramson et al., 2024), for example, with revamped diffusion-based architecture, AF3 has transcended the capabilities of its predecessor by not only predicting protein structures with higher fidelity but also accurately modeling a diverse array of biomolecular complexes. However, it is important to note that AF3 is currently not available as an open-source tool. With the continuous development and in-depth research of AF2 and its derivatives, they are expected to provide broader assistance in theoretical research and direct application in disease diagnosis in the future, becoming more powerful and effective tools for disease diagnosis. We have some ideas, for instance, AF2 can predict the structures and binding interfaces of antigens and antibodies, making it invaluable to the design of immunological assays and detection tools. It can also be utilized to reverse design corresponding antibodies or antigens with high affinity based on the predicted structures. Using AF2’s reverse network, protein sequences corresponding to the designed structures can be predicted (Goverde et al., 2023). Moreover, AF2 can integrate with sequencing technologies to not only detect pathogenic genes but also predict the pathogenic potential of mutations and their impact on biological activities.

During the paper-writing process, we encountered numerous applications of deep learning predictive models such as IgFold (Ruffolo et al., 2023), DeepAb (Ruffolo et al., 2022), and ImmuneBuilder (Abanades et al., 2023). These examples underline the evolving landscape of disease diagnosis, where deep learning models, driven by artificial intelligence, have the potential to facilitate the design of swift and convenient research methodologies.
